# The Cross-Sectional Association between Diet Quality and Depressive Symptomology amongst Fijian Adolescents

**DOI:** 10.1371/journal.pone.0161709

**Published:** 2016-08-25

**Authors:** Rachael Sinclair, Lynne Millar, Steven Allender, Wendy Snowdon, Gade Waqa, Felice Jacka, Marj Moodie, Solveig Petersen, Boyd Swinburn

**Affiliations:** 1 World Health Organization Collaborating Centre for Obesity Prevention, Deakin University, Geelong, Victoria, Australia; 2 School of Health and Social Development, Deakin University, Burwood, Victoria, Australia; 3 Pacific Research Centre for the Prevention of Obesity and Non-Communicable Diseases (C-POND), Fiji National University and Deakin University, Suva, Fiji; 4 IMPACT Strategic Research Centre, Deakin University, Geelong, Victoria, Australia; 5 Deakin Health Economics, Faculty of Health, Deakin University, Burwood, Victoria, Australia; 6 Epidemiology and Global Health Unit, Department of Public Health and Clinical Medicine, Umea University, Umea, Sweden; 7 School of Population Health, Faculty of Medical and Health Sciences, University of Auckland, Auckland, New Zealand; National Cancer Center, JAPAN

## Abstract

**Objective:**

To examine the relationship between diet quality and depressive symptomology amongst a community-based sample of Fijian adolescents.

**Methods:**

Participants included 7,237 adolescents (52.6% girls; mean age 15.6 years) at baseline (2005) and 2,948 (56% girls; mean age 17.4 years) at follow-up (2007/2008), from the Pacific Obesity Prevention in Communities Project. Intervention schools (n = 7) were selected from Nasinu, near Suva on the main Fijian island Viti Levu, and comparison schools (n = 11) were chosen from towns on the opposite, west side of the island. A dietary questionnaire was used to measure diet quality. Factor analysis clustered dietary variables into two unique and independent factors, referred to as healthy diet quality and unhealthy diet quality. Depressive symptomology was assessed via the emotional subscale of the Paediatric Quality of Life Inventory. Both measures were self-reported and self-administered. Multiple linear regression was used to test cross-sectional associations (at baseline and follow-up) between diet quality and depressive symptomology. Variables controlled for included gender, age, ethnicity, study condition, BMI-z scores, and physical activity.

**Findings:**

Strong, positive dose-response associations between healthy diet and high emotional scores (lower depressive symptomology) were found in cross-sectional analyses at baseline and follow-up, among boys and girls. No association was found between emotional health and unhealthy diet.

**Conclusions:**

This study suggests that cross-sectional relationships exist between a high quality diet during adolescence and less depressive symptoms, however more evidence is required to determine if these two variables are linked causally. Trial population health strategies that use dietary interventions as a mechanism for mental health promotion provide an opportunity to further test these associations. If this is indeed a true relationship, these forms of interventions have the potential to be inexpensive and have substantial reach, especially in Low and Middle Income Countries.

**Trial Registration:**

Australian New Zealand Clinical Trials Registry ACTRN12608000345381

## Introduction

The mental health of adolescents continues to be a neglected public health issue, particularly in Low and Middle Income Countries (LMICs). This issue is urgent [1, 2] as, according to current epidemiological estimates, the prevalence of disabling mental illness among adolescents is 20% [3]. One of the most common mental illnesses is depressive disorders with a high global prevalence (estimated at 4–7%) [4, 5]. Depressive disorders (also referred to as internalizing disorders) can take chronic, recurrent, and episodic forms [6], and are recognised by symptomology such as changes in appetite and sleeping patterns, loss of concentration, feelings of worthlessness, sad mood, loss of interest, and irritability [7]. Depressive disorders are associated with serious negative social and wellbeing outcomes including substance abuse, violence, lower educational achievements, and poor sexual health [8]. Depression is also a major risk factor for self-harm, the third leading cause of death amongst adolescents globally [3].

Given the relatively high prevalence, associated high burden, and potential for prevention early in life [9, 10], it is important to explore new and emerging evidence in relation to preventing the onset of mental ill health; for example, the associations between diet quality and depressive symptomology during the adolescent years [11–19]. To date, only one such study has been conducted in a LMIC [11], signifying a substantial gap in our knowledge. It is important to further explore these associations in LMICs as the diets of adolescents in these nations are at different stages of dietary transition (moving from traditional to westernized diets) [20], meaning that results may differ to those seen in High Income Countries (HICs). In addition, studies assessing Health-Related Quality of Life (HRQoL) indicate that adolescents in lower income countries may experience substantially lower HRQoL than their higher income country counterparts [21].

As such, our study explored these relationships in the LMIC nation of The Republic of Fiji. Limited available data suggests that there are some significant mental health issues affecting adolescents in Fiji, with the recent Global School-Based Health Survey (GSHS) finding that 38.9% of students reported feeling sad/depressed every day for 2 weeks in last year, and 17.6% reporting that they had seriously considered suicide during the past 12 months [22].

The objective of this study was to assess the associations between diet quality and depressive symptomology amongst a peri-urban, community-based sample of Fijian adolescents; specifically examining if cross-sectional associations exist. It was hypothesized that a more healthy diet would be associated with lower levels of depressive symptoms, and vice versa.

## Methods

### Participants

The sample included Fijian adolescents from a large peri-urban community participating in the Healthy Youth Healthy Communities (HYHC) Study, part of the Pacific Obesity Prevention in Communities (OPIC) Project (2005–2008) [23–29]. The Pacific OPIC Project aimed to build the capacity of local communities in Australia, Fiji, New Zealand and Tonga to prevent obesity by promoting healthy eating and physical activity (PA). Full details of the Pacific OPIC methods have been published so only the relevant details will be provided [24, 29]. Baseline data were collected from August 2005 to April 2006, with follow-up in May to November 2007 or May to June 2008 [24]. Written informed consent was obtained from schools, parents and the adolescents themselves [24]. The HYHC study was quasi-experimental in design with intervention schools (n = 7) selected from Nasinu, near Suva on the main Fijian island Viti Levu, and comparison schools (n = 11) chosen from towns on the opposite, west side of the island, (for additional information on sampling see Kremer et al. [24] and Swinburn et al. [29]). All adolescents aged 13–18, in class levels 3–6 within selected schools were invited to participate [24].

The original sample size calculation for the Pacific OPIC Project included 1,000 adolescents in each study condition and in each country. According to Tabachnick and Fidell [30], the sample size required for regression analysis is 50 plus 8m where m is the number of predictors; 50+8(10) = 130. The number of participants meets these requirements.

As reported by Kremer et al. [24], there were no intervention effects on diet, as such intervention and comparison schools were merged into one sample, giving 7,237 (out of 9,785; response rate 70%; 3,809 girls and 3,428 boys) at baseline and 2,948 adolescents (out of 7,237; response rate 40%; 1,645 girls and 1,303 boys) at follow-up. Any possible remaining variance attributable to study condition (being in the intervention group) was controlled for by including condition as a covariate.

### Dietary quality

The 83-item English language version of the Adolescent Behaviours, Attitudes and Knowledge Questionnaire (ABAKQ) was used to assess nutrition, physical activity and other health behaviours [24]. A pilot of survey items was undertaken in Fiji to ensure clarity and cultural relevance [24]. This was self-reported and self-administered using Personal Digital Assistants (hand-held computers) [28]. Data reduction of diet quality related questions (n = 22) was conducted through exploratory factor analysis. Two independent factors with eigenvalues greater than one were identified [31, 32]. Rotation did not improve the fit so original factors were used. Factor one, ‘unhealthy diet quality’ included 10 questions from the ABAKQ relating to: consuming takeaway foods for dinner; non-diet soft drinks in the last five school days; chocolates/sweets after school; pies/fried foods/takeaways after school; snack foods after school; purchasing snack foods after school in the last five school days; purchasing meals from a takeaway shop; availability of snack foods at home; availability of chocolates/sweets at home; and availability of non-diet soft drinks at home. Factor two, ‘healthy diet quality’ included 5 questions relating to: availability of fruit at home; daily servings of fruit; daily servings of vegetables; eating fruit after school; and consuming cordial/fruit drinks in the last five school days.

### Depressive symptomology

A subscale of the English version adolescent self-report Pediatric Quality of Life Inventory 4.0 Generic Core Scales (PedsQL^TM^ 4.0) was used to assess depressive symptomology. The 23-item PedsQL^TM^ 4.0, developed by Dr. James W. Varni [33, 34] provides a global HRQoL scale comprising four subscales: physical, emotional, social, and school functioning and wellbeing. The current study utilized the five-item emotional subscale as a proxy measure for depressive symptomology (a continuous variable). This scale has been similarly employed in a previous study [16], and was also piloted in Fiji to ensure cultural relevance and validity [24]. Items are first reversed-scored, then linearly transformed to a 0–100 scale, and finally a mean scale score is estimated conditioned that at least three of the five items are answered [24]. Higher scores signify better mental health [33, 34].

### Covariates

As previously discussed, study condition was included as a covariate. Demographic and anthropometric variables (gender, age in years, and ethnicity) were collected via paper questionnaires. Ethnicity included the categories of Indo-Fijian, Indigenous Fijian or ‘Other’. The ethnic composition of the ‘Other’ category was not collected so may have included many different groups. Because of this lack of detail and small percentage of the sample (n = 167), these participants were dropped from the regression analyses. Height and weight (used to calculate standardised BMI-z scores) were collected by staff trained to use standardised measures and protocols [28]. Four items from the ABAKQ [24, 28] were included as PA indicators: frequency of active transport to and/or from school (number of times active transport was used to and/or from school in the preceding 5 school days); activity levels at recess; activity levels at lunchtime (most common activity at recess and lunchtime, mostly sitting, mostly standing or walking or mostly playing active games); and frequency of after school activities (number of days that active sports/dance/games were undertaken after school over preceding five school days). PA was included as a covariate as associations between higher PA levels and improved mental health have been previously documented in the literature [35].

### Statistical analyses

Cross-sectional analyses were undertaken using Stata version 12.0 (StataCorp, College Station, TX, USA, 2011). Results were significant at p<0.05. The assumptions of multiple linear regression were tested and met. A missing value analysis was undertaken, with very few missing data identified (<5%). Data were deleted case wise in relevant analyses. Univariate outliers (>3 standard deviations beyond the mean) and multivariate outliers (identified using Hadi’s method [36]) were identified and excluded appropriately. All items met the assumptions of normality and independence.

Diet factors were transformed into tertiles to allow testing for possible non-linear and dose-response associations; healthy and unhealthy diet factors were categorised as low (0), medium (1), and high (2) intakes. Differences between baseline and follow-up demographic/anthropometric data were tested using paired t-tests. Multiple linear regression analyses were undertaken at baseline and follow-up to test the strength of associations between the dependent variable (depressive symptomology) and the two independent variables (healthy diet quality and unhealthy diet quality). The initial analyses were unadjusted, while the second analyses included covariates; age, sex, ethnicity, BMI-z scores, PA related variables, and study condition to evaluate the influence of potential confounders. Results were stratified by gender given previous literature indicating that adolescent girls generally experience higher rates of depression compared to boys [37].

### Ethics approval

The HYHC component of Pacific OPIC was approved by the Deakin University Human Research Ethics Committee, Fiji’s National Health Research Committee and the Fiji National Research Ethics Review Committee [24]. The study was also registered with the Australian New Zealand Clinical Trials Registry (ANZCTR), reference number 12608000345381. The use of the HYHC dataset was approved by the local CI, in line with the OPIC protocol. The authors confirm that all ongoing and related trials for this intervention are registered.

## Results

### Sample characteristics

A little over half the sample were girls, and the mean age of participants was 15.6 years at baseline and 17.4 at follow-up (Table 1). At both time points a larger portion of the sample was from the Indo-Fijian ethnic group compared to the Indigenous Fijian group. The proportion of girls in the sample was significantly higher at both baseline (p<0.00) and follow-up (p<0.00). The mean scores for the PedsQL emotional subscale were 64.1 units at baseline and 64.4 at follow-up (Table 1). At baseline, emotional subscale scores were significantly lower amongst girls (60.2, SD 17.2) as compared to boys (68.5, SD 17.0; p<0.00). Similarly in the follow-up sample girls scored significantly lower on the emotional subscale (60.2, SD 15.6) than boys (69.7, SD 15.5; p<0.00). This indicates that girls experienced higher depressive symptomology at both time points as compared to boys.

**Table 1:** Sample characteristics at baseline and follow-up for total sample and by gender.

	Total		Boys	Girls		Boys	Girls	
	Baseline	Follow-up	Baseline			Follow-up		
	N (%)	N (%)	N (%)	N (%)	p	N (%)	N (%)	P
**Total**	7,237	2,948	3,428 (47.4)	3,809 (52.6)	<0.00	1,303 (44.2)	1,645 (55.8)	<0.00
**Ethnicity**								
Indigenous Fijian	3,077 (42.5)	956 (32.4)	1,401 (40.9)	1,676 (44.0)		413 (31.7)	543 (33.0)	
Indo-Fijian	3,794 (52.4)	1,825 (61.9)	1,870 (54.5)	1,924 (50.5)		826 (63.4)	999 (60.7)	
Other	366 (5.1)	167 (5.7)	157 (4.6)	209 (5.5)	<0.00	64 (4.9)	103 (6.3)	0.17
**Study Condition**								
Intervention group	2,670 (36.9)	879 (29.8)	1,399 (36.7)	1,271 (37.1)		406 (31.2)	475 (28.9)	
Comparison group	4,567 (63.1)	2,069 (70.2)	2,410 (63.3)	2,157 (62.9)	0.76	897 (68.8)	1,170 (71.1)	0.18
	**M (SD)**	**M (SD)**						
**Age (years)**	15.6 (1.4)	17.4 (0.9)	15.6 (1.4)	15.6 (1.4)	0.28	17.4 (0.8)	17.3 (0.9)	0.04
**BMI-z score**	-0.01 (1.4)	-0.12 (1.4)	-0.18 (1.4)	0.17 (1.3)	<0.00	-0.24 (1.5)	-0.02 (1.3)	<0.00
**PedsQL–Emotional Subscale**	64.1 (17.6)	64.4 (16.3)	68.5 (17.0)	60.2 (17.2)	<0.00	69.7 (15.5)	60.2 (15.6)	<0.00

Data from the 2005–2008 Pacific Obesity Prevention in Communities Project.

N: Total number at collection point; M: Mean; SD: Standard deviation.

Results significant where p<0.05.

### Cross-sectional associations

Overall, the relationship between diet quality and depressive symptoms remained similar in unadjusted and adjusted models, with healthier diet quality significantly associated with less depressive symptomology (higher emotional subscale scores), whilst unhealthy diet quality was not. Results in fully adjusted models for the associations between healthy diet and depressive symptomology, and unhealthy diet and depressive symptomology are presented in text and in Tables 2 and 3.

**Table 2:** Association between emotional subscale (depressive symptomology) and diet quality factor tertiles for boys at baseline and follow-up.

	Baseline emotional subscale^a^					Follow-up emotional subscale				
	Variable	B	SE	p	95% CI	Variable	B	SE	p	95% CI
**Unhealthy diet quality factor**										
	0 –low					0 –low				
	1 –medium	0.1	1.1	0.96	-2.4, 2.5	1 –medium	-1.4	1.3	0.30	-4.1, 1.3
	2—high	0.7	0.8	0.38	-1.0, 2.5	2—high	-0.9	1.4	0.56	-3.9, 2.2
**Healthy diet quality factor**										
	0 –low					0 –low				
	1 –medium	3.4	0.9	< 0.00	1.4, 5.4	1 –medium	2.3	1.0	0.04	0.2, 4.4
	2—high	7.9	1.0	< 0.00	5.8, 9.9	2—high	5.0	0.9	< 0.00	3.0, 6.9
**Covariates**										
	Age (years)	-0.1	0.3	0.88	-0.7, 0.6	Age (years)	-0.3	0.5	0.51	-1.3, 0.6
	Ethnicity	0.4	0.8	0.58	-1.2, 2.1	Ethnicity	3.1	1.4	0.04	0.2, 6.0
	Condition	1.0	1.1	0.39	-1.4, 3.4	Condition	-2.2	1.0	0.05	-4.3, 0.0
	BMI-z score	0.7	0.2	< 0.00	0.3, 1.2	BMI-z score	0.3	0.3	0.28	-0.3, 0.9
	Walk to school	0.0	0.1	0.99	-0.3, 0.3	Walk to school	0.3	0.1	0.03	0.0, 0.5
	Active at recess	-0.1	0.5	0.84	-1.2, 1.0	Active at recess	-0.5	1.3	0.68	-3.2, 2.1
	Active at lunch	0.2	0.4	0.59	-0.7, 1.1	Active at lunch	2.01	1.0	0.08	-0.3, 4.2
	Active after school	0.4	0.2	0.04	0.0, 0.8	Active after school	0.6	0.4	0.19	-0.3, 1.4

Data from the 2005–2008 Pacific Obesity Prevention in Communities Project.

B: Unstandardized coefficient; SE: Standard error; CI: Confidence interval.

Results significant where p<0.05.

^a^Multiple linear regression model adjusted for age (years); ethnicity; study condition; BMI-z score; and PA (active transport to and/or from school; activity levels at recess; activity levels at lunchtime; frequency of after school activities).

**Table 3:** Association between emotional subscale (depressive symptomology) and diet quality factor tertiles for girls at baseline and follow-up.

	Baseline emotional subscale^a^					Follow-up emotional subscale				
	Variable	B	SE	p	95% CI	Variable	B	SE	p	95% CI
**Unhealthy diet quality factor**										
	0 –low					0 –low				
	1 –medium	-0.3	1.1	0.80	-2.5, 2.0	1 –medium	-1.7	1.2	0.16	-4.2, 0.8
	2—high	-1.0	0.9	0.29	-2.8, 0.9	2—high	-2.1	1.4	0.14	-5.1, 0.8
**Healthy diet quality factor**										
	0 –low					0 –low				
	1 –medium	3.5	0.7	< 0.00	1.9, 5.0	1 –medium	3.0	1.3	0.03	0.4, 5.7
	2—high	6.0	1.0	< 0.00	4.0, 8.0	2—high	4.8	0.9	< 0.00	2.9, 6.7
**Covariates**										
	Age (years)	-0.9	0.3	0.01	-1.6, -0.3	Age (years)	-1.0	0.7	0.16	-2.4, 0.4
	Ethnicity	-1.6	0.9	0.11	-3.5, 0.4	Ethnicity	1.6	1.0	0.15	-0.7, 3.8
	Condition	-1.2	1.2	0.34	-3.7, 1.3	Condition	-1.5	1.2	0.21	-3.9, 0.9
	BMI-z score	-0.2	0.3	0.53	-0.7, 0.4	BMI-z score	1.0	0.4	0.03	0.1, 1.9
	Walk to school	-0.1	0.1	0.58	-0.2, 0.1	Walk to school	0.3	0.1	0.08	-0.01, 0.6
	Active at recess	0.5	0.9	0.55	-1.3, 2.4	Active at recess	3.4	1.1	0.01	1.0, 5.7
	Active at lunch	0.5	0.6	0.47	-0.9, 1.8	Active at lunch	-0.6	1.2	0.64	-3.1, 2,0
	Active after school	0.9	0.7	0.25	-0.7, 2.5	Active after school	-0.2	0.3	0.49	-0.8, 0.4

Data from the 2005–2008 Pacific Obesity Prevention in Communities Project.

B: Unstandardized coefficient; SE: Standard error; CI: Confidence interval.

Results significant where p<0.05.

^a^Multiple linear regression model adjusted for age (years); ethnicity; study condition; BMI-z score; and PA (active transport to and/or from school; activity levels at recess; activity levels at lunchtime; frequency of after school activities).

Dose-response associations were evident at baseline and follow-up. At baseline, compared to those in the low healthy diet group, boys in the medium healthy diet group scored on average 3.4 points higher on the emotional subscale (less depressive symptoms), and those within the high intake group scored 7.9 points higher (Table 2). At follow-up the associations between healthy diet quality and depressive symptomology were in the same direction as baseline and still significant but to a slightly lesser degree.

For girls at baseline, compared to those in the low healthy diet group, girls in the medium group scored 3.5 points higher on the emotional subscale (less depressive symptoms), and those within the high intake group scored 6.0 points higher (Table 3). Similarly, associations between healthy diet quality and depressive symptomology were found for girls at follow-up. Compared to those in the low healthy diet group, those in the medium group scored 3.0 points higher on the scale (less depressive symptoms), and those in the high group scored 4.8 points higher. As for boys, this indicates a dose-response association.

## Discussion

Findings from this study indicated dose-response cross-sectional associations between higher healthy diet scores and less depressive symptoms (higher emotional PedsQL scores), supporting the hypothesis that a healthier quality diet would be associated with lower levels of depressive symptomology. Another study in a LMIC, undertaken in China in 2010, indicated similar results to the present study, suggesting that a traditional (healthy) dietary pattern was associated with experiencing reduced odds of depression symptoms in adolescents [11].

The PedsQL has been tested in a range of countries, and consistently adolescents in LMICs report lower scores as compared to adolescents in HICs [21]. Whilst it is important to consider the impact of socio-economic status (and indeed the developmental status of a country) on this relationship, given that both are established indicators of dietary patterns [38] and mental health outcomes [39], it remains relevant to consider and compare studies conducted in HICs. Several studies conducted in HICs have found associations between higher healthy dietary quality and lower depressive symptomology in adolescents [12, 14, 16, 17, 19], supporting the findings of the present study.

This analysis revealed some indication that the relationships between unhealthy dietary quality and depressive symptomology moved in the hypothesized direction, but the association was not significant. This is contrasted by the Chinese study, which found that those consuming a “snack” (unhealthy dietary pattern) had increased odds for experiencing depression symptoms [11]. Differences in the sample and tools used in the Chinese study as compared to the present study may have contributed to these different findings. Although both studies utilized a factor analysis to identify the natural clustering of the dietary variables, different dietary patterns were identified, suggesting that cultural differences in diet quality and food consumption patterns may influence these associations. Moreover, the Chinese study included more food groups for analysis than the present study. It is also essential to acknowledge that whilst both Fiji and China are categorized as LMICs based on a variety of development indicators according to recent World Bank data [40, 41], both of these countries have very different contexts, which limits comparison as LMIC is not a generalizable term in the context of this research. In addition, other studies in HICs have also found similar associations between unhealthy diets and higher depressive symptoms [11, 13–16, 18] indicating a consistent pattern of associations.

Emotional subscale scores were consistently and significantly lower in girls as compared to boys, indicating that girls experienced higher depressive symptomology. This was not an unexpected finding, given that gender differences for risk of depression in adolescence have been widely acknowledged. The prevalence of depression is considerably higher amongst girls as compared to boys [37], with the rate of depressive symptoms up to three times higher in girls at middle adolescence [42]. However, the difference in emotional subscale scores did not result in substantial difference as to how diet influenced the emotional subscale in each gender, with the emotional subscale changing to a similar extent in both boys and girls at baseline and follow-up.

Overall this study and other literature suggest the presence of a cross-sectional relationship between dietary quality and depressive symptomology [43]. When exploring other study designs, only limited longitudinal studies have been conducted thus far, with conflicting results. Two recent prospective analyses have indicated no longitudinal associations over three-year and two-year follow-up periods respectively [15, 44], whilst another study with a two-year follow-up found the presence of longitudinal associations with healthier diet quality at baseline predicting lower depressive symptoms at follow-up and unhealthy diet quality at baseline predicting lower higher depressive symptoms at follow-up [16]. Null findings longitudinally may relate to a lack of variance in exposures and outcomes, requiring further study.

It is also essential to consider causality and the direction of the relationship. Adolescents with depressive symptoms have been shown to have more perceived barriers to healthy eating [45], whilst those with higher perceived stress have a greater chance of eating less fruits and vegetables [46]. Conversely, this may be a bidirectional association in that depressive symptomology and diet quality could be dependent upon one another. These explanations assume that diet quality and depression are related; however a third, unaccounted variable such as socio-economic factors (e.g. family income, education, social support, and substance addiction) could be the causative predictor of both diet quality and depression, meaning that these variables may not be truly related to one another. However, previous studies have extensively explored potentially explanatory factors, and have reported relationships between diet and depression that are independent of factors such as family structure, education and socio-economic status, social deprivation, alcohol/smoking/drug use, parental conflict, poor family management and social support [14, 15].

A true relationship existing between diet quality and depressive symptomology is further supported by a recent study that utilized daily diary design in a sample of 281 young adults (M = 19.9 years, SD ± 1.2) to assess the direction in which emotional experiences were related to food consumption in a naturalistic setting [47]. Same-day results indicated that on days when participants experienced greater emotional wellbeing, more servings of fruit (p = 0.002) and vegetables (p < 0.001) were consumed [47]. Furthermore, healthy dietary quality drove positive emotional experiences (not positive emotions driving healthy food consumption) as results of lagged analysis indicated that positive emotional experiences were predicted by fruit and vegetable consumption on the previous day [47]. Whilst this research focused on young adults, the same may be true of adolescent populations, warranting further exploration in this age group with a similar study design. Whilst the present study was not designed to measure short time-frame cause and effect, it does suggest that testing lag time between behaviour and outcome is a relevant hypothesis for further work.

### Strengths and limitations

This study was the first to examine the associations between dietary quality and depressive symptomology in Fijian adolescents, and only the second analysis in a LMIC [11]. The study included a large, multi-ethnic sample increasing power of the results [48]. A robust quality of life tool was used that displayed high validity and reliability [33, 34], and was also culturally relevant and meaningful for Fijian adolescents [24]. Furthermore, sufficient dietary indicators were included to enable creating two unique and meaningful diet factors. However, it is acknowledged that dietary items did not capture all food groups, and future studies should include a wider range of dietary items as these may influence results. Reporting errors and response biases could have been present given the self-reported dietary indicators [49]. In addition, the healthy dietary factor included a seemingly unhealthy item; consuming cordial/fruit drinks after school. The research team in Fiji suggested that because cordial was served in such a diluted form, it is generally mostly water. So while it appears unhealthy, it is not compared to all of the other unhealthy diet items, and is not considered to be the same as other sugar sweetened beverages in Fiji.

These results may not be generalizable to all Fijian adolescents (or indeed beyond the Fijian context) as only adolescents from the main island Viti Levu, living in peri-urban communities were included. The major limitation of this study was that as only cross-sectional associations were tested; meaning that determining the causality and the temporal nature of this relationship is limited. Additional studies should explore if there is a true causal relationship between dietary quality and depressive symptomology, and determine the time-course of the effect between diet quality and depressive symptoms.

## Conclusions

There is support for cross-sectional relationships between diet and mental health among adolescents in HICs, and this study revealed similar associations among adolescents in LMICs. However the causality between these two variables remains unknown. Thus there is a great need to study causality using feasible methods. As suggested by Jacka et al. [16], studies that test the effectiveness of dietary modification (promotion of healthy eating at the expense of unhealthy eating) as a means of preventing mental ill-health are required to further explore this association and provide a better understanding of the causal relationships between these variables. Public health strategies that use mental health promotion as a preventative mechanism for depressive symptoms in such a way have the potential to be less expensive and have a greater reach, especially in LMICs, than more traditional psychological interventions such as pharmaceuticals and clinical sessions once depression has already occurred. In addition, this form of intervention would also have a broader impact at a population level as compared to traditional options.

## Supporting Information

S1 Appendix TREND Statement Checklist. (DOCX)

S2 Appendix OPIC study protocol (objectives and designs). (PDF)

S3 Appendix Request for use of data from the OPIC Project. (DOCX)
