# Supplementary material for: The Cross-Sectional Association between Diet Quality and Depressive Symptomology amongst Fijian Adolescents
Source: PLoS One. 2016 Aug 25;11(8):e0161709. doi: 10.1371/journal.pone.0161709 (PMC4999057; doi:10.1371/journal.pone.0161709)
Supplement: S2 Appendix — (PDF) [file pone.0161709.s002.pdf]

The Pacific OPIC Project (Obesity Prevention In Communities) – Objectives and designs

Boyd Swinburn*, Jan Pryor, Marita McCabe***, Robert Carter****, Maximilian de Courten*****, David Schaaf******, Robert Scragg*******

Professor, School of Exercise and Nutrition Sciences, Deakin University, 221 Burwood Highway, Burwood, Victoria 3125, Australia. Email boyd.swinburn@deakin.edu.au ** Dr Fiji School of Medicine, Suva, Fiji. Ph +679 323 3401, email j.pryor@fsm.ac.fj. ***Professor School of Psychology, Deakin University, 221 Burwood Highway, Melbourne, Australia 3125. Email marita.mccabe@deakin.edu.au. ** Associate Professor School of Population Health, 207 Bouverie St, The University of Melbourne, Melbourne, Australia 3010. Email r.carter@unimelb.edu.au. ***** Associate Professor, Department of Epidemiology, Monash University, Commercial Rd, Melbourne, Australia. Email max.decourten@med.monash.edu.au. ******Email d.schaaf@auckland.ac.nz. *******Associate Professor School of Population Health, University of Auckland, Private Bag 92019, Auckland, New Zealand. Email r.scragg@auckland.ac.nz*

Abstract

Background: *Evidence on how to reduce the increasing prevalence of youth obesity is urgently needed in many countries. The Pacific OPIC Project (Obesity Prevention In Communities) is a series of linked studies in four countries (Fiji, Tonga, New Zealand, Australia) which is designed to address this important problem.*

Objectives: *The studies aim to: 1) determine the overall impact of comprehensive, community-based intervention programs on overweight/obesity prevalence in youth; 2) assess the feasibility of the specific intervention components and their impacts on eating and physical activity patterns; 3) understand the socio-cultural factors that promote obesity and how they can be influenced; 4) identify the effects of food-related policies in Fiji and Tonga and how they might be changed; 5) estimate the overall burden of childhood obesity (including loss of quality of life); 6) estimate the costs (and cost-effectiveness) of the intervention programs, and; 7) increase the capacity for obesity prevention research and action in Pacific populations.*

Design: *The community studies use quasi-experimental designs with impact and outcome assessments being measured in over 14,000 youth across the intervention and control communities in the four sites. The multi-strategy, multi-setting interventions will run for 3 years before final follow up data are collected in 2008. The interventions are being informed by socio-cultural studies that will determine the family and societal influences on food intake, physical activity and body size perception.*

Progress and conclusions: *Baseline studies have been completed and interventions are underway. Despite the many challenges in implementing and evaluating community-based interventions, especially in the Pacific, the OPIC Project will provide rich evidence about what works and what does not work for obesity prevention in youth from European and Pacific backgrounds.*

Background

The obesity epidemic is rapidly increasing in both developed and developing countries[1]. Of particular concern is its hold in Pacific populations. The Pacific region has the highest rates of obesity in the world[2], yet the capacity to respond to the epidemic is very limited.

Prevalence rates for overweight and obesity (body mass index, BMI >25kg/m^2) are as high as 75% in Nauru, Samoa, American Samoa, Cook Islands, Tonga and French Polynesia[3]. The Pacific populations living in New Zealand also have extremely high prevalence rates (~80%) compared to the European population (~50%)[4]. The impact of obesity on non-communicable diseases, especially diabetes, is correspondingly enormous and increasing [1] with overweight and obesity ranked as the 7th leading cause of avoidable burden for 2010 and 2020[5].

Of particular concern is its hold in Pacific populations. The Pacific region has the highest rates of obesity in the world,[2] yet the capacity to respond to the epidemic is very limited.

Obesity prevention has, therefore, been recognised as a high priority by the World Health Organization (WHO) in the latest World Health Report[5], successive Pacific Health Forums and other Pacific Consultations[6, 7], and Australian and New Zealand health authorities[8-11] for at least the last 10 years. However, it is only recently that governments have been seeking evidence on what works and does not work for obesity prevention and unfortunately this is very limited. Systematic reviews of the literature have identified less than 30 intervention studies to prevent childhood or adolescent obesity[12, 13]. Most studies have been conducted in primary schools, have been short term and have had modest results at best.

Intervention studies which use optimal health promotion approaches of sustainable, multi-strategy, multi-setting

approaches[14] are, therefore, urgently needed and children and adolescents are obvious priority groups to target because they are still growing in height, they are more responsive to environmental changes, they are a 'captive population' within schools, and society in general has a fundamental responsibility to protect their health and provide healthy environments for them. Pacific children living in New Zealand already have the one of the highest rates of overweight and obesity in the world with a prevalence of about 60% in 5-15 year olds[15]. This contrasts with a much lower rate (about 20%) for contemporary Pacific children living in the islands[16]. However, after these youth in the islands leave school, they are likely to gain about 10-15 kg over 10 years estimated from the current weight difference between decades from recent cross-sectional studies, plus about another 10 kg being the secular trends of whole population weight gain of about 1 kg/year[3, 17].

For these reasons, the Pacific OPIC Project (Obesity Prevention In Communities) is targeting its whole-of-community intervention programs at youth (ages 12-18 years). In addition to determining the effectiveness and cost-effectiveness of the intervention programs, there are several other important environmental areas of research that the Pacific OPIC Project is addressing[18]. The first relates to the socio-cultural aspects of obesity. The beliefs, perceptions, attitudes, values and practices of the society in which one lives have a marked influence on individual behaviours. Identifying the socio-cultural factors that are associated with food and eating, physical activity and sedentary behaviours, as well as body size perceptions which might promote weight gain (ie are 'obesogenic') is fundamental to understanding the drivers of obesity. This information, in turn, can be used to inform the programs and social marketing activities needed to promote healthy eating and physical activity patterns.

The policy environments (legislation, regulations, rules, and policies) also determine behaviours and at a national level these include the trade, agricultural, marketing and fiscal policies as they relate to food. The Pacific Islands have particularly vulnerable food supplies because much of the food is imported.[19] Understanding the influence of these policies on the food supply and evaluating the impact of any policy initiatives is an important component of the Pacific OPIC Project.

The economic dimension of obesity is also extremely important to governments, particularly in the Pacific where the expensive medical and surgical treatment of the complications of obesity, such as diabetes and cardiovascular diseases, is such a large component of the health budget.[20] In addition, obesity and its related diseases reduce life expectancy, productivity, and quality of life – all of which can be counted in the total national burden of obesity.

The stimulus to combine community-based obesity prevention interventions with related socio-cultural, economic and policy studies into a comprehensive program of research across four countries (Fiji, Tonga, New Zealand, Australia) came from an initiative (the International Collaborative for Research Grant scheme, ICRG) by three research funding bodies: the Wellcome Trust (UK), the National Health and Medical Research Council (Australia), and the Health Research Council (New Zealand). The purpose of the ICRG was to link research groups in Australia and New Zealand with others in the Pacific or South East Asia as a way of increasing research capacity in those developing countries on important health priorities. Funding is for 5 years and the Pacific OPIC project was the only one within the ICRG which involved collaborations across New Zealand, Australia and the Pacific.

Objectives and overall design

The objectives of the Pacific OPIC Project are outlined in Table 1. This is an ambitious set of objectives, particularly given the existing low research capacity in the Pacific, the complexity of the task, and the short timelines needed to achieve whole-of-community action and cultural change. In many areas of research endeavour, high income countries are substantially ahead of low income countries in being

Table 1. Objectives of the Pacific OPIC Project in four countries – Fiji, Tonga, New Zealand and Australia

Component	Objectives
**Intervention studies**	**1.** To determine the overall impact of comprehensive, community-based programs on overweight/obesity prevalence in youth
	**2.** To assess the feasibility of the specific intervention components and their impacts on eating and physical activity patterns
**Socio-cultural studies**	**3.** To understand the socio-cultural factors (community attitudes, perceptions, beliefs, values) that promote obesity and how can they be influenced
**Policy studies**	**4.** To identify effects of national and international food-related policies on the supply of foods in Fiji and Tonga and how they might be influenced
**Economic studies**	**5.** To estimate the overall burden of childhood obesity (including loss of quality of life, disease impacts and health system costs) in each country
	**6.** To measure the costs (and cost-effectiveness) of the intervention programs
**Capacity building**	7. To Increase the capacity for obesity prevention research and action in the Pacific

able to answer the key research questions. In relation to obesity prevention, however, countries like New Zealand and Australia might have greater health research and public health expertise than countries like Fiji and Tonga, but the key research questions about how to prevent obesity in adolescents are currently largely unanswered in all countries.

The overall design of the Pacific OPIC Project is outlined in Figure 1. The interventions are the centre pieces and the analytical studies have been chosen to inform or add value to the intervention programs. The specific design features for each of the studies are outlined below.

Community intervention studies overview

Overview: The intervention studies all use a quasi experimental design with an intervention period of 3 years and a cohort follow up. All of the sites take a broad, community-building approach to the interventions and encourage active participation by the community and especially the youth themselves. The details of the development and implementation of the action plans are outlined in an accompanying paper in this journal[21]. There are some variations in the design of the baseline surveys and interventions between sites to accommodate local conditions and constraints (outlined below).

Sample size: The primary outcome variable of interest is changes in body mass index (BMI) with changes in BMI z-score, weight, and percent body fat being closely related secondary outcome variables. In the absence of available data on the standard deviation (SD) of changes in BMI over 3 years in these populations, we used cross-sectional data from a secondary school survey in Auckland which had a high number of Pacific participants (SD for BMI 5.22 kg/m^2, SD for weight 16.8 kg). Assuming a within-person correlation of 0.8, a sample size of 1000 each in the intervention and comparison arms of the study would give sufficient power (ß=0.8, α=0.05) to detect a difference in BMI of 0.41 kg/m2 or 1.3 kg. This was felt to be a reasonable balance between expected effect size and study feasibility and cost. The Auckland study showed no clustering effects by school once ethnicity was controlled for. To allow for dropouts, a target of measuring 1500 participants in each group was set but with the recognition that further recruitment of new entrants to secondary school could be measured in years 2 and 3 of the study to increase the person-years measured. Since Fiji has two large and quite different ethnic groups (Fijians and Indo-Fijians), the study aimed to measure 1500 participants in each ethnic group in both intervention and comparison groups.

Choice of intervention and comparison populations

The criteria for the selection of the intervention populations are outlined in Table 2. Not all criteria were able to be met in each site. Participants from these populations were recruited for the economic, socio-cultural and intervention aspects of the overall study. The choice of comparison group varied by site but, it needed to be as comparable as possible (ethnicity, socio-economic status, likely trajectory of weight gain) and at a distance from the intervention site to minimise contamination.

Participants from these populations were recruited for the economic, socio-cultural and intervention aspects of the overall study.

Community intervention sites

Fiji site: The intervention site chosen was the Nasinu area on the main island of Viti Levu. This is a peri-urban area in

Figure 1. Overall design of the Pacific OPIC Project (PA is physical activity)

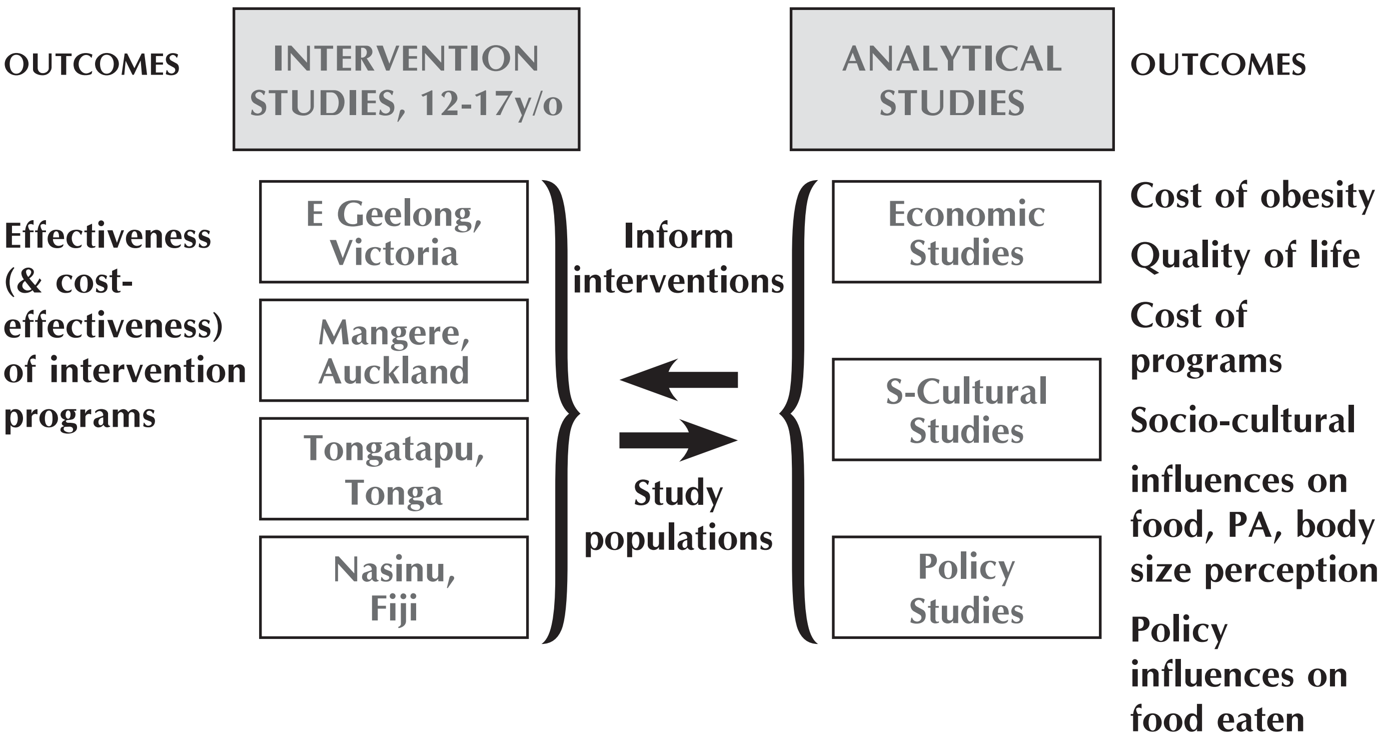

the corridor between Suva and the airport at Nasori. While this area is accessible to Suva and has sufficient numbers of youth who attend schools in the area and sufficient (if not too many) settings for interventions, it has some drawbacks. It is a large area which consists of a number of smaller, coherent communities to which people feel they belong. There are seven main high schools and over 80 churches, mosques, and temples in the intervention area, making it a huge challenge to achieve a high enough 'dose' of intervention across the whole area. The denominator population for the study is adolescents who are in Forms 3-6 at the high schools in the area. Many students living in the Nasinu area travel into Suva to go to school and they are not included in the evaluation, even though they may be exposed to some of the interventions. The comparison population is drawn from eleven schools with a similar mix of Fijian and Indo-Fijian students which are situated in towns on the other (west) side of Viti Levu.

Tonga site: The intervention communities in Tonga are three districts (Nukunuku, Houma, Kolonga) on the main island of Tongatapu and the denominator population is all school students from Forms 2-6 who live in these districts. Many youth, especially in Forms 5 and 6, attend schools outside the districts and this complicated the ascertainment and recruitment of participants. The comparison area is the islands of Vava'u where there are three high schools. Having an intervention site on the main island and the comparison site on the outer islands poses a threat to the validity when comparing the results because the secular trajectory of weight gain in youth in the two sites may be quite different. We will be able to get a sense of this from the weight gain trajectories from previous surveys conducted on these islands. The lower age group used for the Tonga surveys was required because the numbers of eligible youth in the districts were marginal for achieving sufficient power. In the end, fewer than 1000 participants were recruited for each intervention and control site.

Table 2. Criteria for choosing the intervention sites

Component	Criteria
**Community**	• Adequate population size of (accessible) adolescents (for Auckland, a high proportion of Pacific students)
	• Sense of community identity and cohesiveness among community members and organisations
	• Sufficient settings for interventions (schools, churches, community organisations, clubs etc)
	• Presence of 'champions' for change
**Geography**	• Well demarcated boundaries to define denominator population
	• Preferably within a single administrative area
**Access**	• Ease of access for research staff
	• Ease of access for other organisations

New Zealand site: The intervention site in New Zealand is Mangere in South Auckland where data from the Ministry of Education indicated that Pacific students make up a high proportion (59%) of the four high schools in the area. The main Pacific background is Samoan, with Cook Islanders and Tongans being the next largest groups. Two other schools from the South Auckland area were chosen as comparison schools because they had a high proportion of Pacific students (~50%) and were from the same two lowest decile rankings for socio-economic backgrounds of pupils. The denominator population is Years 9-12. In Auckland, the greater ethnic variation may contribute may contribute to a greater design effect, so the sample size is being boosted by measuring the new entrants to the schools (Year 9) in early 2006. Schools and churches are the main intervention settings and, while there is some sense of community within Mangere, a substantial number of Pacific residents attend churches outside the area.

Australian site: In the Barwon-South West region of Victoria (south west coast from Geelong to the South Australia border), a 'Sentinel Site for Obesity Prevention' has been established to support three whole-of-community demonstration projects for obesity prevention in under-5s, primary school-age children and secondary school age children. This last mentioned project is part of the Pacific OPIC Project and the intervention site is located over five schools in the East Geelong / Bellarine region, with the comparison group being a stratified, random selection of schools across the rest of the Barwon-South West region. The population is largely European descent and the average socio-economic status of the area is low. The intervention 'community' does not have a clear demarcation because on its western side it blends in with the rest of Geelong. Also, very few of the youth attend church so the schools will be the dominant settings for interventions. Participants were recruited for the study from Years 7 – 10 (equivalent to the Year levels from the other sites) from each of the schools.

Community intervention measurements

The measurements for the community interventions are shown in Table 3. These have been measured at baseline (2005-6) in the intervention and comparison communities and will be repeated after three years. Students leaving school before 2008 will be assessed prior to leaving school. Baseline questionnaires were programmed into Personal Digital Assistants (PDAs – hand-held mini computers) so that they could be directly filled in by the participants and the information electronically downloaded. Similarly, a program was written so that the body composition data from the bioelectrical impedance scales (Body Composition Analyzer BIA-418, Tanita Corporation, Tokyo, Japan) could

Table 3 Summary of the intervention study evaluation measurements

Component	Measurements	Comments
**Outcomes**	• Anthropometry (height, weight, waist)<br>• Body composition (bioelectrical impedance)	Change in BMI or BMI-z score is the primary outcome but waist circumference or precent body fat may be more sensitive to change
**Impacts**	• Behaviours (eating and physical activity)<br>• Knowledge (indicator questions)<br>• Quality of life (PedsQoL, AQoL2)<br>• Perceptions (body size, role models at home and school)<br>• Environments (school audit)	Impacts relate to action plan objectives. All assessed through questionnaire using standard questions where possible. Audit tool used for school environments, supplemented by youth responses to role model questions.
**Processes**	**Formative evaluation**<br>o Socio-cultural interviews<br>o Development of action plan<br>o Advisory, governance and management structures	Formative processes outlined plus establishing staff, premises etc took about 1 year. Preliminary interviews informed the ANGELO workshop and the action plan development.
	**Process evaluation**<br>o Coordinator reports on activities<br>o Cost data<br>o Minutes, reports, action plans, presentations etc	Coordinator reports are detailed enough to assess implementation issues (reach, uptake, barriers etc) and costs which include financial and time costs.
**Capacity**	• Community Readiness Assessment questionnaire<br>• Follow up stakeholder interviews	Questionnaire at baseline and follow up, supplemented by qualitative findings

BMI is body mass index; PedsQoL is the Pediatric Quality of Life Questionnaire (ref); AQoL2 is the Assessment of Quality of Life questionnaire, version 2 adapted for youth; ANGELO is Analysis Grids for Elements Linked to Obesity;

be electronically downloaded into a laptop. The reason for maximising the electronic data entry (manual data entry was still needed for personal and some demographic data) was to reduce data entry load and errors, simplify the process of data cleaning, and allow for a rapid assessment of the data for analysis and return to the schools and communities

Socio-cultural studies

The groups that participated in the socio-cultural studies were adolescent boys and girls (13-18 years of age) from the following cultural groups: Tongan, Indigenous Fijian, Indo-Fijian, Tongans who reside in Auckland, and Europeans who reside in Barwon-South West region of Victoria. Data were gathered from these five groups at four different stages of the study, utilizing a range of methodologies. These stages are outlined below.

In-depth interviews. The purpose of these interviews was to explore socio-cultural influences on physical activity, eating, and the meaning of body size.

a) ***Preliminary interviews to inform the community workshop.*** Interviews with 6 males and 6 females aged 13 – 17 years from each of the five cultural groups identified socio-cultural factors for inclusion in the community workshop which developed the action plan (called the ANGELO workshop because it used an Analysis Grid for Elements Linked to Obesity for priority setting). They also informed the development of the socio-cultural indicator questions for the baseline questionnaire and the subsequent, semi-structured in-depth interviews. Interviews explored the socio-cultural factors that promoted or protected against weight gain. Interviews were approximately 60-90 minutes and conducted in the language of the participant's choice, audio-taped, transcribed and subjected to content analysis.

b) ***In-depth interviews.*** The purpose of these interviews was to explore socio-cultural influences on physical activity, eating, and the meaning of body size. Messages from parents, extended family, peers, coaches, and the media were explored in depth with 48 participants (24 males, 24 females) in each cultural group. The interviews in Fiji, Tonga and Auckland were conducted by interviewers of the same gender and nationality in the respondents' native tongue. All interviews were translated into English. Interviews were transcribed for analysis. The themes identified in these interviews have been used to inform the action plan and interventions in both Tonga and Fiji.

c) ***The Perceived Socio-cultural Influences on Body Image and Body Change Questionnaire.*** This is a validated scale for use with adolescents that evaluate sources of messages about the body, as well as the nature of these

messages, and their impact on both eating and exercise behaviours. The questionnaire was modified following the in-depth interviews to ensure relevance to each community and will be completed 300 boys and 300 girls in each of the cultural groups.

d) ***Body image distortion studies.*** This method assesses perceptions of the individual's actual and ideal body image. The extent of the discrepancies between what the person would like to look like, and how she/he actually appears, and how accurately she/he perceives his/her body will be assessed. A digital camera projects participants' own bodies onto a computer screen and participants adjust the electronic images on a part-by-part basis to indicate their ideal and perceived actual body. The methodology will be conducted individually with 24 males and 24 females from each cultural group. This phase of the study will determine the discrepancy between each individual's actual and ideal body, and determine how this varies between the different cultural groups.

e) ***Core socio-cultural indicator questions.*** Quantitative socio-cultural questions are included in the baseline and follow up surveys in the intervention and control groups in all sites. These questions will provide quantitative changes over time on the core socio-cultural themes around food and eating, physical activity and inactivity and body size perceptions.

Economic studies

The cost-effectiveness of the four intervention plans will be determined to inform decisions about optimal allocation of resources for obesity prevention. The intervention costing task is a time-consuming one which must be sustained over the three-year intervention duration, involves a large number of players, and has required onsite training and flexible data collection methods. Resource use associated with all intervention activities is documented through a diary approach, and access to records such as invoices, minutes of meetings, staff notes etc. Current practice as reflected by obesity prevention activities in the comparator schools is also being costed. Resources will be valued in terms of local currencies in real prices for the 2005 reference year. Costs will be analysed by expenditure category and key design features to identify cost drivers.

Two quality of life instruments are being administered at baseline and follow-up to facilitate description of the health burden of adolescent overweight and obesity and as an outcome measure in a cost-utility analysis of the interventions. The latter will enable a comparison of the efficiency of obesity prevention with a broader range of health care interventions. As the AQoL-2 (Assessment of Quality of Life) was developed for Australian adults,[22] it has required modification for adolescent use, cultural validation through onsite focus groups at each site, and recalibration of the utility weights. The latter required the completion of ten 'time trade-off' scenarios by samples of 60 adolescents in each site, conducted on a small group basis in the intervention schools. The PedsQL, a pediatric general health profile instrument (module for 13-18 years) is also being used to enhance the credibility of quality of life measurement in the OPIC study[23].

In the final component of the economic studies, the cost and outcome datasets will be brought together with local data on the prevalence of obesity-related diseases and their costs in an economic model that will describe the disease burden and health care cost implications of adolescent obesity. It will also predict the costs and benefits downstream as a result of the interventions through their capacity to reduce obesity-related disease.

Policy studies

The food supply in many Pacific countries is in a vulnerable position because so much is imported and the regulatory environment is not very strong. There are potential problems with food insecurity, food safety, food quality, labelling and food marketing practices[19]. Trade and agriculture policies can have a significant effect on the food supply and there is also the potential for domestic laws to be used to improve the healthiness of the food supply in Pacific countries[24]. The Pacific OPIC project aims to assess policy proposals that could be or will be instituted and to determine their impact on the food supply. Examples of such policies are a potential quota on the importation of mutton flaps and turkey tails into Tonga, the implementation of a 10% tax on soft drinks in Fiji and the institution of a goods and services tax in Tonga.

Trade and agriculture policies can have a significant effect on the food supply and there is also the potential for domestic laws to be used to improve the healthiness of the food supply in Pacific countries[24].

Capacity building in Pacific research

In the Pacific, six staff on the OPIC project or from the Fiji School of Medicine are undertaking postgraduate studies (mainly Postgraduate Diploma in Public Health) and have used the OPIC study for their research unit. Staff in Fiji and Tonga have been able to participate in four workshops on social marketing, two on epidemiology and statistics using Epiinfo, and others on project management, writing papers, and health promotion. Several Pacific staff also presented their data at conferences – the Pacific Medical Association conference in Tonga, 2005 and the Community-based Obesity Prevention conference in Geelong, 2006. Investigator meetings are usually held three times a year in Fiji or Tonga and these are used as an opportunity to upskill the Pacific team members in aspects of research.

In New Zealand, five Pacific graduate students, funded by the OPIC project, are currently enrolled to complete PhD theses or Masters of Public Health degrees at the Pacific research centre at the University of Auckland. These students, along with a Pacific principal investigator, will form the nucleus of a Pacific health research centre at the School of Population Health and have the capacity to lead the development of research on issues affecting the health of the Pacific community in New Zealand.

The research activities for the Pacific OPIC project which are based in Australia include the Geelong intervention site, the socio-cultural, economic, and policy analyses and these involve Deakin University, The University of Melbourne and Monash University. These increased research links between these Victorian universities and the Pacific countries may continue to contribute to capacity building in the Pacific beyond the life of the project.

Progress to mid 2006

The formative stages of the project included the identification of the intervention sites and the engagement of the communities, recruiting staff and establishing the research and administrative structures, development and testing of research instruments, undertaking the preliminary socio-cultural interview, training staff, undertaking an ANGELO workshop and developing the draft action plan. This took at least a year, sometimes longer, to implement from mid 2004. Baseline data collection started at various times within 2005 in each of the sites and was completed by mid 2006 in all sites. The analytical studies started in 2005 and will continue through to 2008.

The health promotion activities started in 2005, but again this varied by site because of the competing activities of the baseline measurements and the different levels of health promotion capacity in each country. Training programs in health promotion and social marketing were included for staff and each site capitalised on the surveys and start of interventions to launch their projects and to gain some publicity for them. The background to the health promotion activities and the action plans are outlined in more detail in the paper by Schultz et al.

Large, community-based intervention programs come with many challenges, mainly due to their complexity, the multiple partners involved, the substantial capacity building needs, and the time needed to orient all the related organisations towards common goals.

Lessons learned to date

Even at these early stages, many lessons have been learned along the way. Large, community-based intervention programs come with many challenges, mainly due to their complexity, the multiple partners involved, the substantial capacity building needs, and the time needed to orient all the related organisations towards common goals. The lead times are long and the efforts needed to create the trust and partnerships are substantial, but in the end, it is these relationships which provide the backbone for the programs and their sustainability. Partners need to take the time to understand each others' agendas and to have the flexibility to be able to create the maximum synergies for the community while minimising the organisational politics and barriers.

Champions who are influential within the community, organisations, and governments are also crucial. They can create those vital visions and aspirations that inspire people to make changes, and they can open the doors to decision-makers and pave the way for progress.

The complexity of a whole-of-community intervention program is further layered by the research and evaluation components. Each community is different, and this requires substantial flexibility in designing the evaluation and managing the burden and rigour of the measurements. There are many trade-offs between what would be ideal for science and what suits the community. For example, schools are heavily constrained in the time they can allocate to the assessment process, since this takes up the time of staff and students. Often a balance needs to be found between these realities and the tightness required of the scientific assessment. There is a tendency for scientists to over-measure and so questionnaires get expanded, sample sizes get increased, measurement frequencies increase, measurements become more detailed, and so on. It is a high risk in these types of programs that the efforts involved in measurement (including all the ethics applications for each component) outweigh the efforts put into the interventions.

These challenges for intervention and research are more than doubled in Pacific countries.

The capacity for health promotion and research in the Pacific is already low (mainly due to limited financial resources and few trained health professionals in the area) and these projects stretch those resources even further. Unlike Australian and New Zealand research institutions, the Pacific institutions like the Fiji School of Medicine get no linked overheads and infrastructure costs for research from government.

Conclusions

The Pacific OPIC project is a large, complex health promotion and research endeavour across four countries. The partnership model between tertiary institutions in Pacific, New Zealand and Australia allows substantial, high quality research projects to be conducted in Pacific countries where the existing public health and research capacities are low. The outcomes of the Pacific OPIC project will guide future obesity prevention efforts in all four countries, and this will be particularly important in the Pacific region where obesity prevalence rates are the highest in the world and obesity complications are a huge burden on health care resources.

Acknowledgements

The authors would like to thank the many people involved in the Pacific OPIC project: including other co-investigators, other staff and postgraduate students, partner organisations and especially the schools and their pupils.

References

1. WHO. Obesity: preventing and managing the global epidemic. Report of a WHO consultation. Geneva: WHO; 2000. Report No.: WHO Technical Report Series 894.
2. Hodge AM, Dowse GK, Zimmet PZ, Collins VR. Prevalence and secular trends in obesity in Pacific and Indian Ocean island populations. Obesity Research 1995;3(Suppl 2):77s-87s.
3. Coyne T. Lifestyle diseases in pacific communities. Noumea, New Caledonia: Secretariat of the Pacific Community; 2000.
4. Russell D, Parnell W, Wilson N. NZ food, NZ people: Key results of the 1997 National Nutrition Survey. In: 1997 National Nutrition Survey. Wellington: Ministry of Health, New Zealand; 1997.
5. WHO. World Health Report 2002: Reducing Risks, Promoting Healthy Life. Geneva: World Health Organization; 2002.
6. WHO. Plan of Action for the Western Pacific Declaration on Diabetes 2000-2005. Manila, Philippines: World Health Organization, Western Pacific Region; 2001.
7. WHO. Workshop on obesity prevention and control strategies in the Pacific. Manila, Philippines: WHO, Regional Office for the Western Pacific; 2000 October, 2000.
8. National Health and Medical Research Council. Acting on Australia's weight. Canberra: National Health and Medical Research Council: Commonwealth of Australia; 1997.
9. Thomas M, Wise M. Healthy Weight Australia. A National Obesity Strategy.: The Australasian Society for the Study of Obesity; 1995.
10. National Obesity Taskforce. Healthy Weight 2008 - Australia's Future. Canberra: Department of Health & Ageing; 2003.
11. Pumau O, Kai O. Healthy Action - Healthy Eating. Towards an Integrated Approach to Physical Activity, Nutrition and Healthy Weight for New Zealand. Wellington, New Zealand: Ministry of Health; 2002 2002.
12. Summerbell C, Waters E, Edmunds L, Kelly S, Brown T, Campbell K. Interventions for preventing obesity in children. Cochrane Database Syst Rev 2005(3):CD001871.
13. Doak C, Adair LS, Monteiro C, Popkin BM. Overweight and underweight coexist within households in Brazil, China and Russia. J Nutr 2000;130:2965-2971.
14. Swinburn B, Egger G. Preventive strategies against weight gain and obesity. Obes Rev 2002;3(4):289-301.
15. Parnell W, Scragg R, Wilson N, Schaaf D, Fitzgerald E. New Zealand Food, New Zealand Children: Key results of the 2002 National Nutrition Survey. Wellington: Ministry of Health, New Zealand; 2003.
16. Fukuyama S, Inaoka T, Matsumura Y, Yamauchi T, Natsuhara K, Kimura R, et al. Anthropometry of 5-19-year-old Tongan children with special interest in the high prevalence of obesity among adolescent girls. Ann Hum Biol 2005;32(6):714-23.
17. Ministry of Health. Fiji Non-communicable diseases (NCD) STEPS survey 2002. Suva, Fiji Islands: Ministry of Health; 2002.
18. Swinburn B, Egger G, Raza F. Dissecting obesogenic environments: the development and application of a framework for identifying and prioritizing environmental interventions for obesity. Prev Med 1999;29(6 Pt 1):563-70.
19. Lawrence M. An analysis of the appropriateness, acceptability and implications of regulatory approaches to control the flow of fatty foods in Pacific Island countries. Background paper prepared for the FAO/SPC/WHO Pacific Islands Food Safety and Quality Consultation, Nadi, Fiji, 11-15 November 2002.
20. WHO. Health care decision-making in the Western Pacific Region. Diabetes and the care continuum in the Pacific Island Countries: WHO, Regional Office for the Western Pacific; 2003.
21. Schultz J, Utter J, Mathews L, Cama T, Mavoa H, Swinburn B. Action Plans and Interventions - The Pacific OPIC Project (Obesity Prevention In Communities). Pacific Health Dialog 2006.
22. Richardson J, Day N, Peacock S, Lezzi A. Measurement of the quality of life for economic evaluation and the assessment of quality of life (AQoL) Mark 2 instrument. The Australian Economic Review 2004;37(1):62-88.
23. Varni JW, Seid M, Rode CA. The PedsQL: measurement model for the pediatric quality of life inventory. Med Care 1999;37(2):126-39.
24. Lawrence M. Using domestic law in the fight against obesity: An introductory guide for the Pacific. Melbourne: Deakin University; 2002.
