# Supplementary material for: The Cross-Sectional Association between Diet Quality and Depressive Symptomology amongst Fijian Adolescents
Source: PLoS One. 2016 Aug 25;11(8):e0161709. doi: 10.1371/journal.pone.0161709 (PMC4999057; doi:10.1371/journal.pone.0161709)
Supplement: S3 Appendix — (DOCX) [file pone.0161709.s003.docx]

**REQUEST FOR USE OF DATA FROM THE PACIFIC OPIC PROJECT**

**Please send completed form to the designated in-country investigator from which data use is being requested:**

**Fiji:**

Dr Ilisapeci Kubuabola

Email: [ilisapeci.kubuabola@fnu.ac.fj](mailto:ilisapeci.kubuabola@fnu.ac.fj)

Post: Co-ordinator C-POND (Pacific Research Centre for the Prevention of Obesity and Non-communicable Diseases), Research Unit, Fiji School of Medicine, Tamavua campus, College of Medicine, Nursing & Health Sciences, Fiji National University, Suva, Fiji

Tel: +679 3233258

**Part 1 Conditions under which data access will be permitted**

1.1 That data will be used solely for the purpose stated in Part 3.

1.2 That permission for use of data and dissemination of any materials arising from the data is signed by the in-country Principal Investigator, Co-Investigator or his/her nominee

1.3 That data be co-analysed and/or co-interpreted by a person from each cultural group being subjected to analysis

1.4 That all co-analysts and co-interpreters are co-authors

1.5 That study participants and teams who collected, entered and managed and/or analysed data be acknowledged in writing in all written documents, presentations and publications resulting from data use

1.6 That the Pacific OPIC Project at all of the relevant sites is acknowledged whenever the data are used

1.7 That the designated in-country investigator gives clearance to submit a paper containing results for that country for journal review

1.8 That the designated in-country investigator be provided with a copy of all publications (student projects and theses, published papers, conference presentations) arising from data use

**Part 2 Requestor Details**

Surname: _______________________________________________________________________

Forenames: _____________________________________________________________________

Address: ___________________________________________________________________________

______________________________________________________________________________________________________________________________________________________

Phone number: _________________

Email address: ______________________________________

Organization/Institution name and address: __________________________________________________________________________

______________________________________________________________________________________________________________________________________________________

Student ID (if applicable) _____________________________________________________

Supervisor in the case of students:

Name: _________________________________________

Designation: _________________________________

Programme of Study (if relevant) _____________________________________________________

___________________________________________________________________________

Date: _____________________________

**Part 3 Requested Data**

3.1 Description of requested data set/s: _____________________________________________________________________________________________________________________________________________________________________________________________________________________

3.2 Country of origin of data requested: __________________________________________________

3.3 Period within which data requested were collected: _____________________________________________________________________________________________________________________________________________________________________________________________________________________

3.4 Sole purpose for which data will be used: ___________________________________________________________________________________________________________________________________________________________________________________________________________________________________________________________________________________________

3.5 Proposed dissemination plan (include conferences and proposed papers): _____________________________________________________________________________________________________________________________________________________________________________________________________________________

3.6 Request recommended by

NB This section for completion if applicant is not an OPIC team member

Full name _______________________________________

Designation ______________________________________

Professional relationship to person requesting data _______________________________________________________

Signature __________________________

Date: __________________________

**Part 4 Approval Statement**

4.1 I, the requestor, agree to abide by all the conditions stated in Part 1

Full name: ____________________________________________________________

Signature: _____________________________________________________________

4.2 Permission granted by

Full name: ____________________________________________________________

Position in the Project: ___________________________________________________

Signature: ________________________________________

Date: ________________________________

**Part 5 Accessing the data**

Once approval has been received, all data may be accessed by contacting:

Nicholas Crooks

E-mail: nicholas.crooks@deakin.edu.au

Post: Geelong Waterfront Campus, Locked Bag 20001, Geelong, VIC, 3220, Australia

Tel: +61 3 524 79330
